# Prevalence and determinants of food addiction among Palestinian university students: a cross-sectional study

**DOI:** 10.1007/s40519-025-01803-7

**Published:** 2025-12-26

**Authors:** May Hamdan, Fatima Al-Amouri, Mohammed Motasem Jaber, Dana Marbu, Mohammad Taleb Abed, Areej Halayqa, Balqess Al-Zabadi, Eman Qawasmeh, Roua Shaheen, Manal Badrasawi

**Affiliations:** 1 Department of Health Professions, Program of Healthy and Therapeutic Nutrition/Faculty of Medicine, Palestine Polytechnic University, Hebron, Palestine; 2 Department of Nutrition and Food Technology, College of Medicine and Health Sciences, An-Najah National University, Nablus, 44839 Palestine; 3 Department of Medicine, College of Medicine and Health Sciences, An-Najah National University, Nablus, 44839 Palestine; 4 Department of Nutrition and Dietetics, Faculty of Pharmacy, Nursing and Health Professions, Birzeit University, West Bank, Palestine

**Keywords:** Food addiction, Yale Food Addiction Scale (YFAS), Mental health, Dietary behaviors, Palestine

## Abstract

**Background:**

Food addiction (FA) is characterized by an insatiable urge to consume high-calorie, sugary, hyper-palatable foods beyond energy needs. This condition is associated with having obesity, binge eating, and comorbid physical, psychological, and social complications. While FA shares characteristics with other eating disorders, it is still understudied in many populations, especially university students. This study aims to assess the prevalence of FA among Palestinian university students and identify associated nutritional, social, psychological, and lifestyle factors.

**Methods:**

A cross-sectional study was conducted on university students using a random sampling technique. A paper-based structured questionnaire was used to collect data related to sociodemographic, medical history, FA using the Yale Food Addiction Scale (YFAS), and mental health using the Depression Anxiety and Stress Scale-21 (DASS-21). Dietary behaviour was assessed using the Mediterranean Lifestyle Index (MEDLIFE), while Body Mass Index (BMI) was used to evaluate weight status. The assessed lifestyle habits included quality of sleep using the Sleep Hygiene Index (SHI), physical status using the International Physical Activity Questionnaire (IPAQ), and smoking status. Data were analyzed using one-way ANOVA and Chi-square tests and multiple linear regression.

**Results:**

The study involved 1435 participants, mostly female (66.2%), single (91.0%), and nonsmokers (71.1%). The study found that 79% of participants had no FA, 18% had mild addiction, and 3% had moderate to severe addiction. There was a significant relationship between smoking, chronic disease, SHI, or previous surgery and FA.

However, there was no association between FA and other sociodemographic factors, medical history, lifestyle characteristics, or nutritional characteristics. The multiple linear models found that age (*B* = 0.080), anxiety (*B* = 0.120), depression (*B* = 0.154), SHI (*B* = 0.225), BMI (*B* = 0.153), Mediterranean diet (*B* = 0.106), and previous surgery (*B* = − 0.064) are significant predictors of FA risk at *p* ≤ 0.001.

**Conclusion:**

Our study identified a notable presence of FA among university students. Key factors associated with FA included smoking, chronic disease, history of surgery, higher BMI, and adherence to the Mediterranean diet, as well as psychological factors such as stress, anxiety, depression, and poor sleep hygiene. These findings elaborate on the complex relationship of physical, psychological, and lifestyle factors contributing to FA. Further research is recommended to clarify the causal relationships behind these associations.

*Level of evidence*: level Ⅳ

**Plain English summary:**

Food addiction (FA), a disorder defined by an insatiable need to consume high-calorie, sugary, and highly appealing meals more than energy requirements, is linked to having obesity, binge eating, and other medical, psychological, and social issues. The purpose of this study is to determine the prevalence of FA among Palestinian university students, as well as the dietary, social, psychological, and lifestyle aspects that contribute to it. The study discovered a 21% FA prevalence in the sample group, with 18% having mild FA and 3% suffering from moderate to severe addiction. These rates are greater than in the overall population but lower than in previous research including university students. The study discovered a link between FA and a variety of variables, including smoking, chronic disease, a history of surgery, and a higher BMI. Individuals with a history of chronic illness and surgery were likely connected to FA, possibly due to the psychological impact of long-term illness and body image concerns. A Mediterranean diet was associated with lower levels of FA. Stress, anxiety, sadness, and poor sleep hygiene were all also increasing the likelihood of FA. These findings emphasize the diverse character of FA, which is influenced by biological, psychological, and environmental factors, as well as the need for additional research to investigate these complicated relationships.

## Introduction

Food addiction (FA), or addiction-like eating behavior, is a disorder characterized by an insatiable urge to consume high-caloric, high-sugar, hyper-palatable foods that surpass what is needed to sustain a stable energy level for daily functioning [1]. FA affects 20% of the general population, with a considerable increased incidence in females compared to males in the population of those aged < 35 years compared to their counterparts [2], with the exclusion of college students, of whom the incidence was observed to reach 24% of the population [3]. Also, higher incidence rates of FA have been established among individuals with obesity and/or binge eating behavior [4].

Food addiction is not yet recognized in the Diagnostic and Statistical Manual of Mental Disorders, Fifth Edition (DSM-5), but it shows many similarities with substance abuse disorders [5]. The classification of food addiction (FA) as a substance-related addiction is substantiated by literature indicating that some behaviors associated with the consumption of refined foods meet the criteria for substance use disorders. The hypothesis is supported by observational and empirical data. A new diagnostic classification and therapy strategies for modifying overeating and FA-related behaviors may result from this theory [6]. Individuals who are diagnosed to have FA manifest as a spectrum of behaviors overlapping with substance use disorders and binge-eating or bulimia nervosa, such as craving, impulsivity, reduced control, altered reward sensitivity, and the repeating episodes of consuming large portions of food and mood improvement after consumption [7, 8]. FA can be classified as a substance use disorder or behavioral addiction based on a set of characteristics such as hunger, taste, enjoyment, food function, loss of social ties, weight issues, and awareness of the illness [8]. Food ingestion triggers these effects by causing a surge in dopamine, which in turn produces pleasurable feelings [9].

People who experience symptoms associated with food addiction may be at increased risk for additional health conditions, as these symptoms can affect physical, psychological, and social well-being. Food addiction is often associated with higher body weight [10] and eating disorders, leading to a high risk of metabolic disorders and cardiovascular disease [11]. Furthermore, this condition negatively impacts the patient's social well-being and interactions with their environment, resulting in social isolation and a deterioration of both abnormal eating habits and food addiction symptoms [12].

Food addiction has been reported to be more common in the 18–29 age range, and it is well known that young people are greatly influenced by the food environment in which food is consumed for pleasure or because of prior positive experiences [13]. Furthermore, compared to other age groups, young adults, especially those at the universities, are more likely to develop a food addiction because they may be living on their own without parental supervision or monitoring, which can lead to looser boundaries or restrictions and make them more susceptible to experiencing addictive behaviors, including food intake [14]. However, the onset of FA and its associated factors remain unclear among Palestinian university students. This study aimed to evaluate the prevalence of FA and outlines the factors that might be associated with it, including physical, social, psychological, and lifestyle characteristics among Palestinian university students.

## Methodology

### Study design and settings

This cross-sectional study was used to determine the prevalence of FA among university students in Palestine. The study encompassed participants from three Palestinian universities: labeled as A, B, and C. The study included undergraduate participants within the age of 18 to 23 years. Conversely, employees, faculty members, postgraduate or pregnant students were excluded from the study. Exclusion criteria included individuals with current eating disorder diagnosis or medical conditions which might influence food intake.

### Sample size

The sample size was calculated using G Power software with an alpha of 0.05 (two-sided) and 80% power. The effect size was determined using the prevalence of FA among university students from a similar study; the study found the prevalence of FA was 10.1% using The Yale Food Addiction Scale (YFAS) [1]. The required sample size is 140 participants for each sex, indicating 280 participants. Furthermore, the sample size calculation took into account the primary objective of the study; determining the relationship between FA and metal health, a moderate effect size of Cohen's d of 0.5, a level of significance or type I error of 0.05 (5%), and a power or type II error (1-β) of 0.8 (80%). The sample size calculations all indicated that a minimum of 480 individuals would be sufficient to conduct the analyses. Given the dropout (20%) due to missing data or exclusion criteria, the sample size was increased to include at least 580 participants from each sex. The required sample size is 1160 participants.

### Sampling and data collection approach

A stratified convenience sampling approach was used to recruit the participants. Data collection was completed over five months, from July to November 2022. Following ethical approval and permission from the three participating universities, the study was announced to the students within their respective departments. Students were approached on campus and invited to participate voluntarily after confirming that they met the study inclusion criteria. They were verbally briefed about the purpose of the study and were given the written consent forms to sign before they started answering the pre-designed, validated, paper-based questionnaire through face-to-face interviews. While participants were recruited from three universities, selection within each university was based on convenience rather than random sampling, and the final numbers from each university reflect differences in participation rates and logistical access during data collection.

The questionnaire lasted for 10 to 13 min and was completed independently. However, further assistance was available from the research team. The questionnaire consisted of 6 sections. These encompassed sociodemographic data, medical history, detection of FA experiences, mental health assessment, dietary behaviors, and some lifestyle habits. Study tools that were not originally available in Arabic were translated using forward–backward translation by bilingual specialists. The final version of the questionnaire was checked for content validity by experts in the field prior to data collection.

### Study tool

The study used the following instruments and tools to establish operational definitions for the study variables.

### Sociodemographic data

Sociodemographic data included multiple-choice questions regarding age, sex, area of living (city, village, or camp), living status (with spouse and family, with relatives, student housing, or other), number of family members, year of study at the university (1st, 2nd, 3rd, 4th, or 5^th^ year), marital status (married, single, or other), and family income (< 1500, 1500–3000, 3000–5000, or > 5000 NIS).

### Medical data

Medical history information involved dichotomous (yes/no) questions, including whether the participants have any chronic disease, such as hypertension, diabetes, asthma, and allergies; take certain medications on a regular basis; and have had any types of surgeries in their lives.

### Lifestyle behaviors

This section assessed three habits: smoking status, physical activity, and sleep quality. Participants were asked to self-report their smoking status (yes or no), smoking duration, and smoking type (cigarette or shisha). Furthermore, the Sleep Hygiene Index (SHI) was used to assess the participants' sleep quality. The SHI consists of 13 self-rated questions with a response option in a 5-point Likert-type scale, ranging from 0 (indicating “Never”) to 4 (indicating “Always”), and the total score is generated through summation of each item score. The maximum obtainable score of the SHI is 52; higher scores indicate worse sleep hygiene. The Arabic SHI has a good internal consistency, with a Cronbach’s alpha of 0.749 [15]. In this study, the Cronbach’s alpha coefficients was 0.724.

The short version of the International Physical Activity Questionnaire (IPAQ-SF) was utilized to analyze participants' physical activity levels. The IPAQ-SF is a 7-item questionnaire. In the first six questions, participants were asked about the frequency and daily cumulative time of physical activity in the past seven days, from the aspects of high-intensity PA, moderate-intensity PA, and walking. The last question asked about the sitting situation [16, 17]. The results were categorized into three levels: low activity, moderate activity, or high activity levels. IPAQ-SF has been consistently shown to have a high reliability (ranging from 0.66 to 0.88) [18].

### Food addiction

FA has not been recognized as a clinical disorder in the fifth edition of the Diagnostic and Statistical Manual (DSM-5) of Mental Disorders [19]. The Yale Food Addiction Scale (YFAS) stands as the sole established instrument for FA diagnosis. Notably, the YFAS was updated in 2016 and has been translated into numerous languages globally [20]. The Arabic version of the modified Yale Food Addiction Scale (mYFAS 2.0) was applied in the current study. The scale is available as a short screening measure to assess FA. It consists of 13 items; 9 questions are core, with 1 question from each of the symptom groups that compose the seven diagnostic criteria, plus two individual items that assess the presence of clinically significant impairment and distress. Scored on an 8-level Likert scale ranging from 0 (indicating never) to 7 (indicating every day). If a participant reported ≥ 3 of the seven diagnostic dependence symptoms with clinical significance, they met the criteria for FA. FA was categorized according to the number of symptoms associated with clinical significance criteria into mild (symptom count score 2–3), moderate (symptom count score 4–5), and severe (symptom count score 6–11). The mYFAS 2.0 reported good internal consistency (Cronbach’s *α* = 0.89), good test–retest reliability (ICC = 0.69) [21]. In this study, internal consistency of the mYFAS was good, with Cronbach’s alpha coefficients of 0.872.

### Nutritional characteristics

Participants were asked to self-report their weight and height, and BMI was calculated and categorized as being underweight: under 18.5 kg/m^2^, having normal weight: greater than or equal to 18.5 to 24.9 kg/m^2^, having overweight: greater than or equal to 25 to 29.9 kg/m^2^ and having obesity: greater than or equal to 30 kg/m^2^ [22].

The analysis of participants' dietary patterns utilized the Mediterranean Lifestyle Index (MEDLIFE). Comprising twenty-two items, including food consumption (15 items) and dietary habits (7 items). Each item is scored between 0 (negative) and 1 point (positive). The cumulative analysis generates a score ranging from 0 (indicating the least adherence) to 22 (indicating the best adherence). The score then was categorized as low (0–9), medium (10–13), and high adherence (14–22) [23, 24].

### Mental health

The health status of the university students was measured using the Depression Anxiety and Stress Scale-21 (DASS-21). It is a short version of 42 questions that includes 21 questions designed to assess three negative emotions, which are depression, anxiety, and stress. Response options are on a 4-point scale (0 = did not apply to me at all and 3 = applied to me most of the time). Higher scores indicate more psychological distress; the final score of each item group was multiplied by two [25, 26]. In this study, internal consistency of the DASS-21 was excellent, with Cronbach’s alpha coefficients of 0.919.

### Statistical analysis

Data were analyzed using IBM SPSS version 25. Descriptive statistics were used to describe the quantitative variables, while categorical variables were described in percentages and frequencies. A one-way ANOVA test was used to investigate the relationship between continuous variables and FA. A Chi-square test was used for analyzing the association between FA and categorical data. Confidence intervals (Cls) for differences were calculated, and a level of 5% was set for considering the tests significance. Lastly, a multivariate analysis was carried out using multiple linear regression. All the variables that showed significant association with FA in the univariate analysis (*p* < 0.05) were included in the model.

## Results

### Sociodemographic factors of the participants

A total of 1435 participants were enrolled in our study. Females were the major sex, and the majority were single (Table 1). The mean age of participants was 19.9 ± 1.4 years, and the mean number of family members was 6.5 ± 2.2. Participants were divided between universities, as follows: 518 (36.1%) from university A, 481 (33.5%) from university B, and 436 (30.4%) from university C.

**Table 1:** Sociodemographic characteristics

Variable (unit)		*n* (%)
Sex	Male	484 (33.8)
	Female	950 (66.2)
Area of living	City	827 (57.7)
	Village	536 (37.4)
	Camp	72 (5.0)
Living status	With spouse and family	1367 (95.3)
	With relatives	10 (0.7)
	Student housing	48 (3.3)
	Others	10 (0.7)
Year of study	1st year	254 (17.7)
	2nd year	410 (28.6)
	3rd year	327 (22.8)
	4th year	365 (25.4)
	5th year	79 (5.5)
Marital status	Single	1305 (91.0)
	Married	90 (6.3)
	Other	40 (2.8)
Family income (NIS)	< 1500	62 (4.4)
	1500–3000	258 (18.2)
	3000–5000	499 (35.2)
	5000 and above	600 (42.3)

NIS: New Israeli Shekel (1 NIS is equivalent to 0.27 US Dollars)

### Medical history and lifestyle characteristics

Most of participants were nonsmokers; 17.8% had surgery, and a minority have chronic disease, with diabetes being the most reported, and they took medication with Trafen (tranexamic acid 500 mg + mefenamic acid 250 mg) and metformin, which were the most reported. The mean total of the Sleep Hygiene Index was 22.1 ± 8.5 (Table 2).

**Table 2:** Medical history and lifestyle characteristics

Variable		*n* (%)
Smoker	Yes	414 (28.9)
	No	1021 (71.1)
Physical activity	Sedentary	453 (31.6)
	Moderate	564 (36.9)
	Highly active	368 (25.6)
Chronic disease	Yes	53 (3.7)
	No	1382 (96.3)
Surgery	Yes	256 (17.8)
	No	1179 (82.2)
Medications	Yes	76 (5.3)
	No	1359 (94.7)

### Nutritional characteristics

Table 3 shows that our participants were mostly categorized as being either underweight or normal weight, with the majority having low adherence of the Mediterranean diet.

**Table 3:** Nutritional characteristics

Variable (unit)		*n* (%)
BMI (kg/m^2^)	Underweight (< 18.5 kg/m^2^)	136 (34.0)
	Normal weight (18.5–24.9 kg/m^2^)	957 (45.2)
	Overweight (25–29.9 kg/m^2^)	264 (20.7)
	Obese (above 30 kg/m^2^)	65 (9.6)
Mediterranean diet adherence	Low	481 (67.3)
	Medium	639 (18.6)
	High	293 (4.6)

BMI: body mass index

### Mental health characteristics

Participants showed a total mean stress score of 17.8 ± 10.1, a total mean anxiety score of 15.5 ± 9.9, and a total mean depression score of 16.6 ± 10.1.

### Prevalence of food addiction

**Fig. 1:**
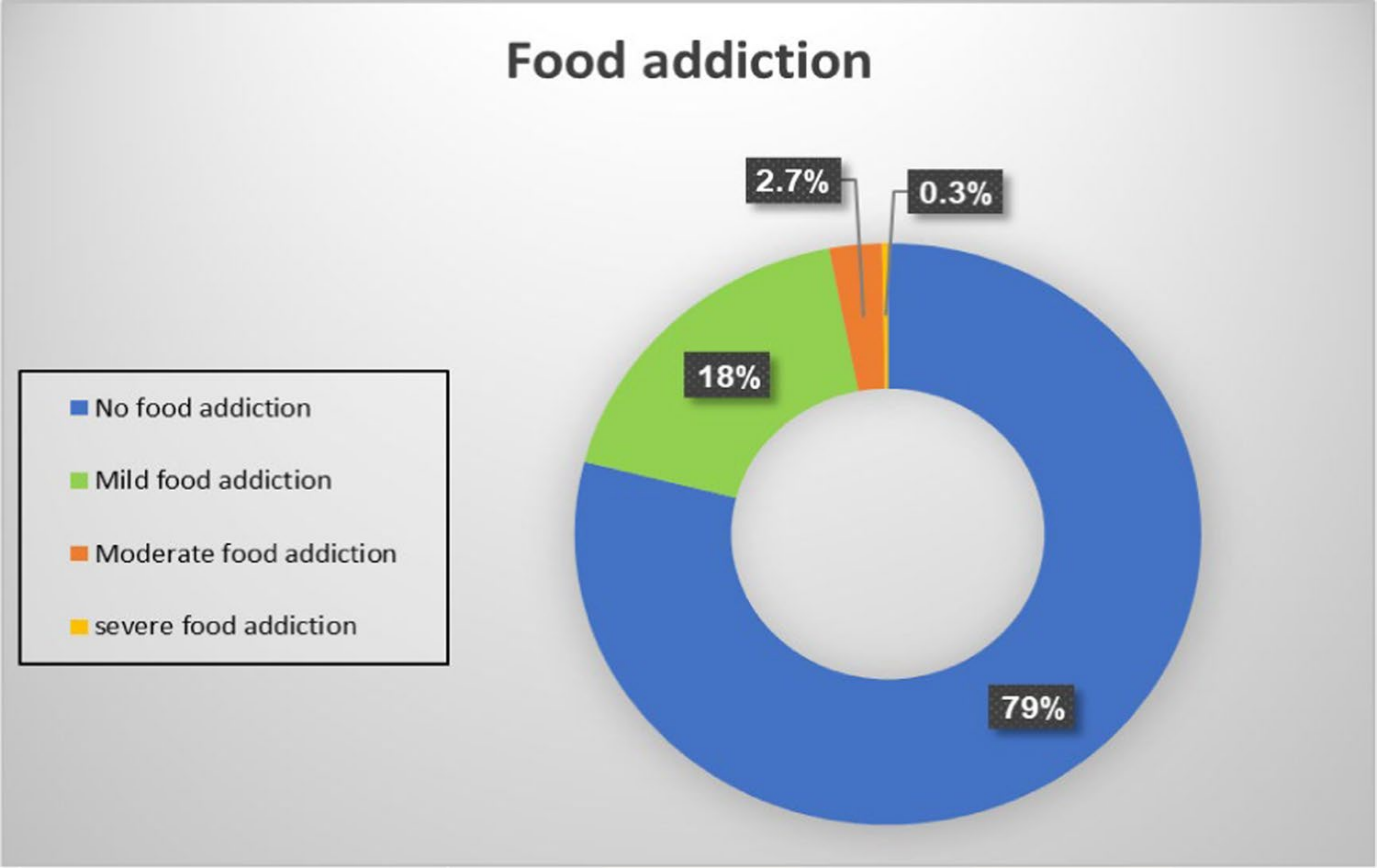
Food addiction categories prevalence

The mean score of food addiction symptoms is 2.3 ± 2.6 (0–11), which is considered mild FA symptoms. Figure 1 showed that 79% of our participants did not experience food addiction symptoms, and 18% were with mild addiction; also, 3% were with moderate to severe addiction. The mean and standard deviation scores of food addiction based on symptom classifications were as follows: No food addiction; the mean ± SD is 0.7 ± 0.5, ranging from 0 to 1.9, the mild category is 2.7 ± 0.5, ranging from 2 to 3.92, while the moderate and severe category is 4.7 ± 0.7, ranging from 4 to 7.

### Associations of food addiction with sociodemographic factors

The study found no association between FA and sociodemographic factors, as shown in Table 4.

**Table 4:** Association of food addiction with sociodemographic factors

Category		Food addiction n (%)			*P*-value
		No FA	Mild FA	Moderate FA	
Sex	Female	766 (80.6)	156 (16.4)	28 (2.9)	0.095
	Male	369 (76.2)	102 (21.1)	13 (2.7)	
Area of living	City	658 (79.6)	147 (17.8)	22 (2.7)	0.494
	Village	426 (79.5)	95 (17.7)	15 (2.8)	
	Camp	51 (71.8)	16 (22.5)	4 (5.6)	
Living status	With spouse and family	1088 (79.6)	240 (17.6)	39 (2.9)	0.045^a^
	With relatives	5 (50.0)	4 (40.0)	1 (10.0)	
	Student housing	38 (79.2)	9 (18.8)	1 (2.1)	
	Others	5 (50.0)	5 (50.0)	0 (0.0)	
Marital status	Single	1040 (79.7)	230 (17.6)	35 (2.7)	0.389
	Married	65 (72.2)	20 (22.2)	5 (5.6)	
	Other	31 (77.5)	8 (20.0)	1 (2.5)	
Family income (NIS)	< 1500	46 (74.2)	13 (21.0)	3 (4.8)	0.883
	1500–3000	209 (81.0)	42 (16.3)	7 (2.7)	
	3000–5000	397 (79.6)	87 (17.4)	15 (3.0)	
	5000 and above	471 (78.5)	113 (18.8)	16 (2.7)	
Year of study	1st year	216 (19)	32 (12.4)	5 (12.2)	0.193
	2nd year	327 (28.8)	73 (28.3)	10 (24.4)	
	3rd year	248 (21.8)	67 (26)	12 (29.3)	
	4th year	287 (25.3)	69 (26.7)	9 (22)	
	5th year	57 (5)	17 (6.6)	5 (12.2)	

^a^chi-square statistic was invalid due to incomparable data set

### Associations of food addiction with medical history and lifestyle characteristics

We found that there was a significant relationship between smoking, having a chronic disease, or having a previous surgery with FA (*p* = 0.011), (*p* = 0.001), and (*p* ≤ 0.001), respectively. No relationship with taking medication was found, as presented in Table 5.

**Table 5:** Association of food addiction with medical history

Category		Food addiction n (%)			*P*-value
		No FA	Mild FA	Moderate FA	
Smoker	Yes	309 (74.6)	87 (21.0)	18 (4.3)	0.011*
	No	827 (81.0)	171 (16.7)	23 (2.3)	
Physical activity	Sedentary	348 (76.8)	93 (20.5)	12 (2.7)	0.092
	Moderate	445 (78.9)	95 (16.8)	24 (4.3)	
	Highly active	300 (81.2)	63 (17.1)	5 (1.4)	
Chronic disease	Yes	37 (69.8)	11 (20.8)	5 (9.4)	0.010*
	No	1099 (79.5)	247 (17.9)	36 (2.6)	
Previous surgeries	Yes	172 (67.2)	70 (27.3)	14 (5.5)	0.000*
	No	964 (81.8)	188 (15.9)	27 (2.3)	
Medication	Yes	56 (73.7)	19 (25.0)	1 (1.3)	0.205
	No	1080 (79.5)	239 (17.6)	40 (2.9)	

^*^Significant at *P* < 0.01 according to the chi-square test

### Associations of food addiction with nutritional characteristics

We found that there was a strong, significant relationship between participants BMI categories and adherence to the Mediterranean diet with FA (*p* ≤ 0.001) and (*p* ≤ 0.001), respectively (Table 6).

**Table 6:** Association of food addiction with nutritional characteristics

Category		Food addiction n (%)			*P*-value
		No FA	Mild FA	Moderate FA	
BMI (kg/m^2^)	Underweight	116 (85.3)	17 (12.5)	3 (2.2)	0.000*
	Normal weight	782 (81.7)	155 (16.2)	20 (2.1)	
	Overweight	179 (67.8)	71 (26.9)	14 (5.3)	
	Obese	49 (75.4)	12 (18.5)	4 (6.2)	
Mediterranean diet adherence	Low (0–9)	404 (84.0)	62 (12.9)	15 (3.1)	0.000*
	Medium (10–13)	506 (79.2)	117 (18.3)	16 (2.5)	
	High (14–22)	206 (70.3)	77 (26.3)	10 (3.4)	

BMI: body Mass Index

^*^Significant at *P* < 0.01 according to the chi-square test

### Relationship of food addiction with age, mental health and lifestyle

There were significant differences in the means of age, stress, anxiety, depression, and SHI for FA (Table 7).

**Table 7:** Relationship of food addiction with age, mental health and lifestyle presented in mean ± SD

Variables	Food addiction			F (CI)	*P*-value
	No FA (*n*) mean ± SD	Mild FA (*n*) mean ± SD	Moderate FA (*n*) mean ± SD		
Age (year)	19.9 ± 1.4 (1136)	20.1 ± 1.3 (258)	20.5 ± 1.3 (41)	8.2 (19.85–19.99)	0.000*
Stress	16.6 ± 10.0 (1123)	22.2 ± 8.9 (256)	22.7 ± 9.8 (41)	39.0 (17.24–18.29)	0.000*
Anxiety	14.3 ± 9.7 (1122)	20.3 ± 9.4 (256)	20.7 ± 8.9 (41)	47.7 (15.02–16.05)	0.000*
Depression	15.2 ± 9.8 (1122)	21.8 ± 9.4 (245)	21.9 ± 10.1 (41)	54.9 (16.02–17.08)	0.000*
SHI	20.9 ± 8.6 (1131)	26.1 ± 7.9 (257)	28.5 ± 8.8 (41)	55.2 (21.64–22.52)	0.000*

SHI: sleep hygiene index. Stress, anxiety and depression scores are according to DASS-21

^*^Significant at *P* < 0.01 according to the one-way ANOVA test

### Food addiction predictors

Variables included in the multivariate regression model were primarily selected based on statistical significance (*p* < 0.05) from univariate analyses (age, stress, anxiety, depression, SHI, BMI, Mediterranean diet, smoking, chronic disease, and previous surgery). The multiple linear regression model explained approximately 21.3% of the variance in food addiction scores (*R*^2^ = 0.213), indicating that these factors collectively accounted for a modest proportion of the variability in the outcome. After controlling for potential confounders, many variables remained statistically significant, including age, anxiety, depression, SHI, BMI, the Mediterranean diet, and previous surgery were significant predictors of FA risk (*p* < 0.05), as detailed in Table 8.

**Table 8:** Food addiction predictors

Factors	P-value	Standardized (B)	CI	*P*-value
Age (year)	0.001*	0.080	(0.026–0.106)	0.000*
Stress	0.796	− 0.011	(− 0.010–0.008)	
Anxiety	0.001*	0.120	(0.005–0.022)	
Depression	0.000*	0.154	(0.008–0.026)	
SHI	0.000*	0.225	(0.023–0.037)	
BMI (kg/m^2^)	0.000*	0.153	(0.029–0.056)	
Mediterranean diet	0.000*	0.106	(0.019–0.050)	
Smoking	0.578	0.014	(− 0.087–0.155)	
Chronic disease	0.916	0.003	(− 0.269–0.300)	
Previous surgery	0.009*	− 0.064	(− 0.332–0.048)	

^*^p < 0.05 according to multiple linear regression

## Discussion

We found that 21% of the study sample of university students fulfill the criteria of food addiction, distributed as mild FA among 18% and moderate to severe FA among 3%, which is considered higher than other community groups, yet lower than the same group in other studies [2]. For instance, in Brazilian university students, using mYFAS, FA prevalence was 19.1% [27], 12.9% among undergraduate students from Northwest Mexico [28], and 11.4% among Egyptian university students [29], which can be attributed to the differences in the social and environmental factors.

In this study, there was a significant relationship between FA and smoking, which is similar to other studies that found that food addiction is more common among smokers, which is attributed to the effect that smoking and the consumption of food that is listed to be addictive, exert on the brain, both stimulating the reward pathway [30].

Furthermore, the study found that the presence of a chronic disease and previous history of surgery were associated with FA, which can be in the light of existing literature attributed to the effect a chronic disease can have upon the affected patients, like the distortion of body image in the case of apparent disease-related manifestation of the external appearance of the patient, and due to the psychological burden of the disease, both these factors increase the risk of the development of the disordered eating behaviors [31, 32]. On the other hand, literature has revealed that patients are prone to developing FA symptoms post-surgery, especially after bariatric surgery [33]. However, due to the lack of data on the specific types of chronic diseases and surgeries, the study was unable to explore their direct relationship with FA risk.

In addition, the study found a correlation between patients’ BMI, having obesity with FA; this comes in agreement with previous literature, which demonstrated that having obesity was a result of various factors, including the behavior of overeating in response to different food cues like smell and taste. This result is aggravated by the effect that palatable foods exert on the brain mesocorticolimbic circuits, which reinforces the urge to overconsume these foods, promoting obesity and further intertwining this relationship [34].

Moreover, the study found a significant relationship between adherence to the Mediterranean diet and FA. Previous studies on the topic have suggested that adherence to the Mediterranean diet may be linked to reduced consumption of hyper-palatable food [35]. However, this was not directly assessed in this study; thus, further research is required in this regard.

A significant relationship between FA and age, stress, anxiety, depression, and sleep hygiene. Previous studies have elaborated that age plays a critical role in the development of FA, especially in college students which is attributed to the sudden changes in the eating and dietary habits of college students, which mainly consist of unhealthy food [36]. Adult students transitioning from school to university experience difficulties adhering to healthy habits due to lack of time and stressors and skip meals, eat unhealthy snacks, dine out, and consume fast food. In addition, they tend to exhibit a deficit of knowledge about healthy diet options, which influence their eating patterns and nutrition [37], all of which explains the influence of eating habits on FA among the young adult age group.

The study also found that stress correlates with FA. This is in line with previous findings, which indicated that elevated levels of stress lead to defects in the reward system in the brain, leading to increased appetite for hyper-palatable foods as an aversive response [38]. Additionally, the study found a correlation between depression and FA, which is discussed in existing literature and attributed to the dopaminergic activity in response to depression [39]. Studies have shown that patients who suffer from depression may exhibit impulsive actions toward food resembling FA; this is further worsened by the short effect of this behavior leading to relapsing of this behavior [40]. Also, The study showed a relationship between anxiety and FA, which is seen more commonly in females than males, and the reason for this behavior is how each gender is affected by anxiety and how they respond [41, 42].

The study found that sleep hygiene is correlated with FA, which is explained by the effect of sleep deprivation on the corticosteroid projections, which leads to their wakening and results in an increased consumption of hyper-palatable foods. Similar behavior is seen among patients with substance abuse disorder, where in these patients the lack of sleep leads to impairment in their cognitive functioning, leading to difficulties in stopping their behaviors that may be labelled as addictive [43].

## Strengths and limitations

The research's positive aspects include a large sample size, a comprehensive questionnaire covering a wide range of factors, and the use of widely utilized, validated, and reliable tools. The study has significant limitations. The use of a cross-sectional approach complicates determining the causal relationship between risk factors and FA. The data were collected using self-report questionnaires, which may be susceptible to memory bias and consequently impact the results. In addition, a significant limitation of the study is the lack of detailed data on the types of chronic diseases and previous surgeries reported by participants, which makes it challenging to determine their direct relationship with FA risk. Nevertheless, it’s important to consider that the use of a stratified convenience sampling approach may limit the generalizability of the findings, as participants were not randomly selected. Consequently, the results may better represent students who were accessible and willing to participate, rather than the entire university student population.

## Conclusion

Food addiction was prevalent among 21% of the study population, with 18% and 3% experiencing mild and moderate to severe FA, respectively. The study found a significant relationship between FA and several factors, including smoking, chronic disease, history of surgery, and higher BMI. Having obesity and FA were found to be interconnected as well. In addition, adherence to a Mediterranean diet was associated with lower rates of FA. Stress, anxiety, depression, poor sleep hygiene, and younger age also showed strong correlations with FA. These findings underscore the multifaceted nature of FA, influenced by biological, psychological, and environmental factors, and highlight the need for further research to explore this complex relationship. In addition, these findings emphasize the need for targeted interventions that address both nutritional and mental health aspects for university students. Furthermore, routine screening for disordered eating patterns by promoting adherence to balanced dietary patterns, such as the Mediterranean diet, which lower the risk of FA and related health issues. From a broader perspective, implementation of nutritional education programs and food policy within university settings could further prevent FA, encourage healthy and balanced food choices, and promote overall student well-being.

## Data Availability

The dataset supporting the conclusions of this article is available upon request from the corresponding author.

## Abbreviations

FA: Food addiction
SHI: Sleep hygiene index
IPAQ-SF: The short version of the international physical activity questionnaire
DSM-5: The fifth edition of the Diagnostic and Statistical Manual
YFAS: The Yale Food Addiction Scale
mYFAS 2.0: The modified Yale Food Addiction Scale
BMI: Body Mass Index
MEDLIFE: The Mediterranean Lifestyle Index
DASS-21: The depression anxiety and stress scale-21
NIS: New Israeli Shekel
